# Pharmacological and Psychosocial Interventions in Patients with Limb Amputations in Sri Lanka: a Case Series

**DOI:** 10.1192/j.eurpsy.2026.12014

**Published:** 2026-07-31

**Authors:** C. Costa, N. Fonseka, P. Vidyatilake, C. Wijesinghe

**Affiliations:** 1 Colombo North Teaching Hospital, Colombo, Sri Lanka; 2 Albury Wodonga Health, NSW, Australia

## Abstract

**Introduction:**

Limb amputation is often followed by phantom limb pain and grief -both of which frequently go underdiagnosed in busy clinical settings. These difficulties compound physical disability and hinder recovery (Flor et al., Lancet Neurol 2002;1:182–189; Bhayana et al., Injury 2024;55:111828). While phantom limb pain has been extensively studied globally, the psychosocial impact of limb loss, particularly grief, has not been systematically assessed in Sri Lanka since the stigma associated with expressing grief, often discourages patients from seeking help. Integrating pharmacological and psychosocial interventions in a multidisciplinary framework enhances recovery and well-being.

**Objectives:**

To examine the clinical profiles, psychosocial challenges, and treatment outcomes of patients with limb amputations managed at the Pain Management Clinic, Colombo North Teaching Hospital, Sri Lanka.

**Methods:**

Methods:

Five patients (3 males, 2 females; aged 31–61 years) with limb amputations were reviewed at the Pain Management Clinic, Colombo North Teaching Hospital. Purposive sampling was used. Data were collected via structured questionnaire and clinical records. Management included pharmacotherapy and psychotherapy. Pain was rated on a numerical scale before and after intervention. Quantitative data were analyzed using descriptive statistics, paired t-test, Wilcoxon signed-rank test, and Cohen’s d; qualitative data were thematically analyzed.

**Results:**

All patients reported phantom limb pain with associated grief, anger, sleep disturbance, or depressive symptoms. Pre-treatment pain ranged 5–9/10 (mean 7.1 ± 1.1). Interventions included neuropathic agents (e.g., pregabalin, amitriptyline, carbamazepine, sertraline) and psychotherapies (CBT for pain, grief counselling, mirror therapy, relaxation, family therapy). Post-treatment scores ranged 1–5/10 (mean 3.2 ± 1.6), reflecting a significant reduction (p = 0.013; Cohen’s d = 1.91).

Patients also reported improved mood, coping, and social functioning. This is the first documented assessment in Sri Lanka of grief related to limb loss measured pre- and post-grief therapy in amputees.

**Image 1:**
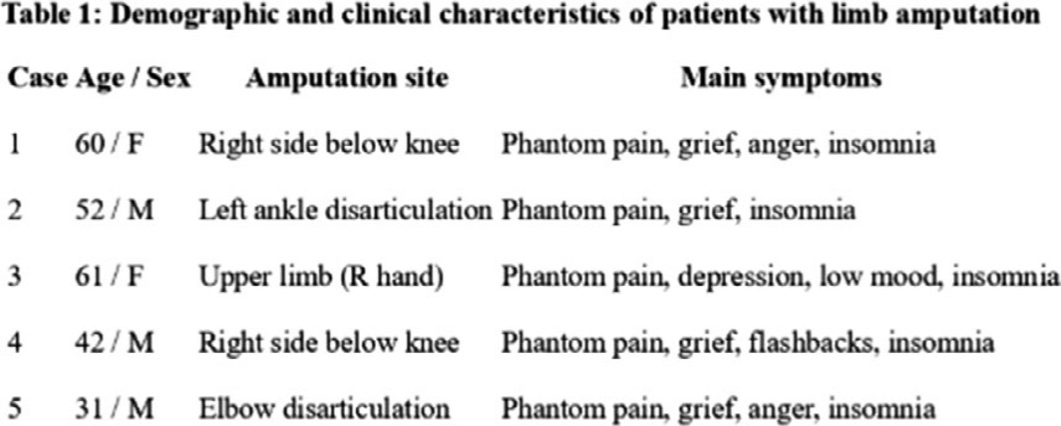
Image 1: Long description.

**Image 2:**
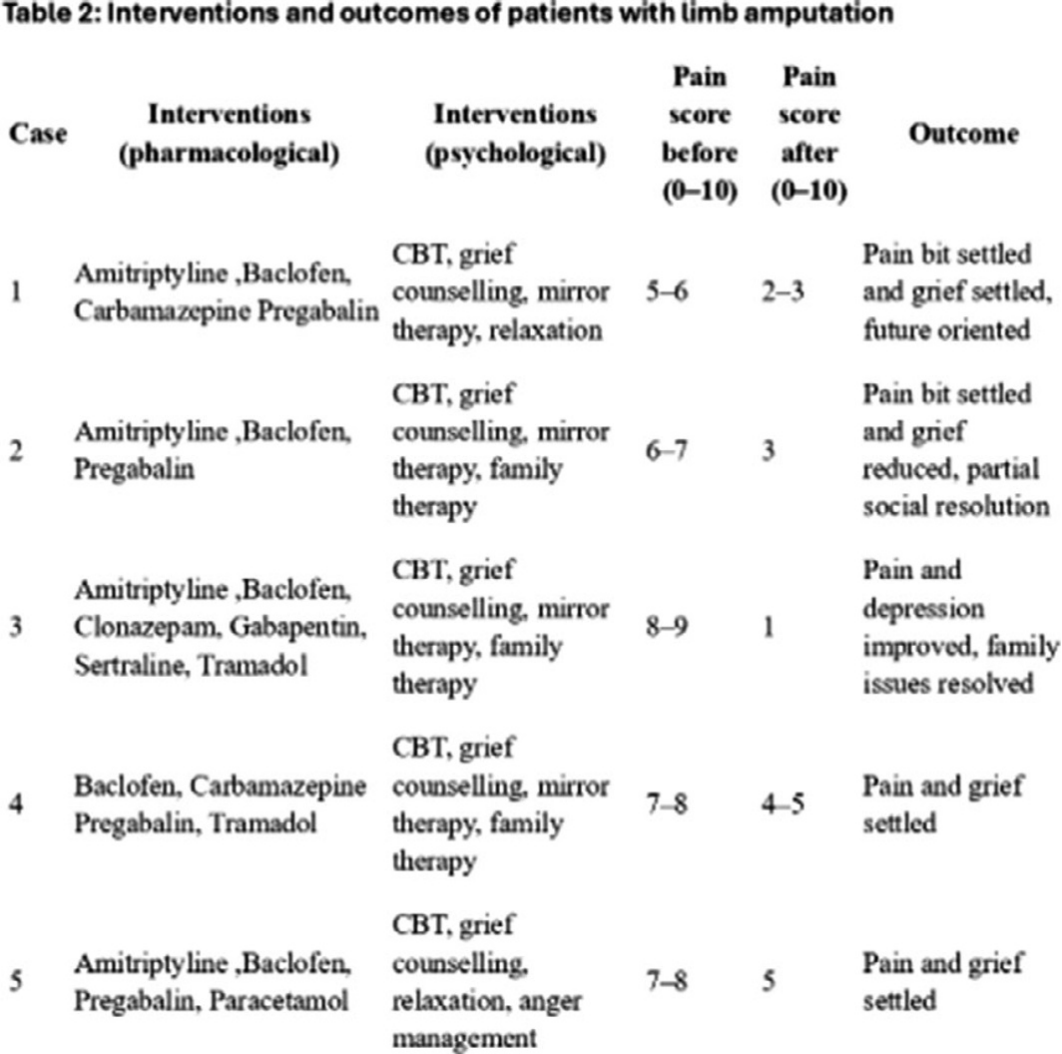
Image 2: Long description.

**Image 3:**
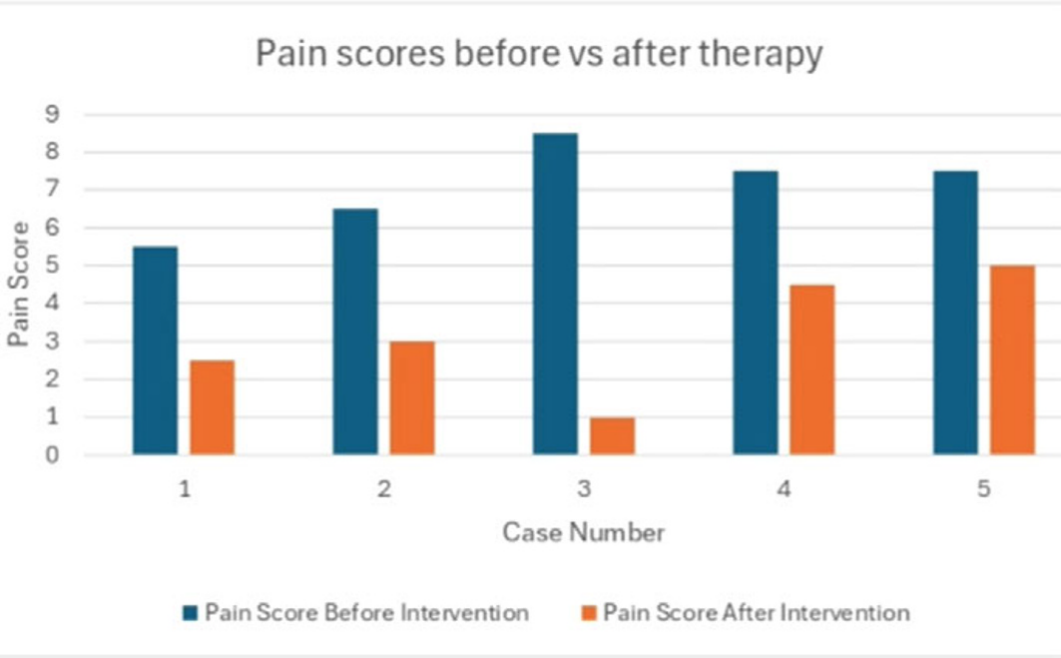
Image 3: Long description.

**Conclusions:**

This case series highlights the importance of integrating pharmacological and psychosocial interventions in managing amputees, while underscoring the value of proactively addressing grief related to limb loss, even when patients do not openly express it. The novelty of this Sri Lankan study aligns with the global evidence base, though larger controlled studies are needed to clarify the relative contributions of individual interventions.

**Disclosure of Interest:**

None Declared

## Long descriptions

### Image 1: Long description

The table contains four columns: Case, Age / Sex, Amputation site, and Main symptoms.

* Case 1: 60 / F. Amputation site is Right side below knee. Main symptoms are Phantom pain, grief, anger, insomnia.

* Case 2: 52 / M. Amputation site is Left ankle disarticulation. Main symptoms are Phantom pain, grief, insomnia.

* Case 3: 61 / F. Amputation site is Upper limb (R hand). Main symptoms are Phantom pain, depression, low mood, insomnia.

* Case 4: 42 / M. Amputation site is Right side below knee. Main symptoms are Phantom pain, grief, flashbacks, insomnia.

* Case 5: 31 / M. Amputation site is Elbow disarticulation. Main symptoms are Phantom pain, grief, anger, insomnia.

Navigate back to Image 1:.

### Image 2: Long description

The table consists of six columns: Case, Interventions (pharmacological), Interventions (psychological), Pain score before (0–10), Pain score after (0–10), and Outcome.

* Case 1: Pharmacological interventions include Amitriptyline, Baclofen, Carbamazepine, and Pregabalin. Psychological interventions include C B T, grief counselling, mirror therapy, and relaxation. Pain score decreased from 5–6 to 2–3. Outcome: Pain bit settled and grief settled, future oriented.

* Case 2: Pharmacological interventions include Amitriptyline, Baclofen, and Pregabalin. Psychological interventions include C B T, grief counselling, mirror therapy, and family therapy. Pain score decreased from 6–7 to 3. Outcome: Pain bit settled and grief reduced, partial social resolution.

* Case 3: Pharmacological interventions include Amitriptyline, Baclofen, Clonazepam, Gabapentin, Sertraline, and Tramadol. Psychological interventions include C B T, grief counselling, mirror therapy, and family therapy. Pain score decreased from 8–9 to 1. Outcome: Pain and depression improved, family issues resolved.

* Case 4: Pharmacological interventions include Baclofen, Carbamazepine, Pregabalin, and Tramadol. Psychological interventions include C B T, grief counselling, mirror therapy, and family therapy. Pain score decreased from 7–8 to 4–5. Outcome: Pain and grief settled.

* Case 5: Pharmacological interventions include Amitriptyline, Baclofen, Pregabalin, and Paracetamol. Psychological interventions include C B T, grief counselling, relaxation, and anger management. Pain score decreased from 7–8 to 5. Outcome: Pain and grief settled.

Navigate back to Image 2:.

### Image 3: Long description

The y-axis is labeled Pain Score with a scale from 0 to 9. The x-axis is labeled Case Number with categories 1 through 5. A legend at the bottom indicates that dark blue bars represent Pain Score Before Intervention and orange bars represent Pain Score After Intervention.

* Case 1. Before score is 5.5 and after score is 2.5.

* Case 2. Before score is 6.5 and after score is 3.

* Case 3. Before score is 8.5 and after score is 1.

* Case 4. Before score is 7.5 and after score is 4.5.

* Case 5. Before score is 7.5 and after score is 5.

Across all five cases, the orange bars are consistently lower than the blue bars, indicating a universal decrease in reported pain scores after therapy.

Navigate back to Image 3:.

